# Transcriptomic effects of propranolol and primidone converge on molecular pathways relevant to essential tremor

**DOI:** 10.1038/s41525-022-00318-9

**Published:** 2022-08-04

**Authors:** Charles-Etienne Castonguay, Calwing Liao, Anouar Khayachi, Yumin Liu, Miranda Medeiros, Gabrielle Houle, Jay P. Ross, Patrick A. Dion, Guy A. Rouleau

**Affiliations:** 1 Department of Human Genetics, McGill University, Montreal, QC Canada; 2 Montreal Neurological Institute, Department of Neurology and Neurosurgery, McGill University, Montreal, QC Canada; 3 Faculté de Médecine, Université de Montréal, Montreal, QC Canada

**Keywords:** Movement disorders, Molecular medicine

## Abstract

Essential tremor (ET) is one of the most common movement disorders, affecting nearly 5% of individuals over 65 years old. Despite this, few genetic risk loci for ET have been identified. Recent advances in pharmacogenomics have previously been useful to identify disease related molecular targets. Notably, gene expression has proven to be quite successful for the inference of drug response in cell models. We sought to leverage this approach in the context of ET where many patients are responsive to two drugs: propranolol and primidone. In this study, cerebellar DAOY and neural progenitor cells were treated for 5 days with clinical concentrations of propranolol and primidone, after which RNA-sequencing was used to identify convergent differentially expressed genes across treatments. Propranolol was found to affect the expression of genes previously associated with ET and other movement disorders such as *TRAPPC11*. Pathway enrichment analysis of these convergent drug-targeted genes identified multiple terms related to calcium signaling, endosomal sorting, axon guidance, and neuronal morphology. Furthermore, genes targeted by ET drugs were enriched within cell types having high expression of ET-related genes in both cortical and cerebellar tissues. Altogether, our results highlight potential cellular and molecular mechanisms associated with tremor reduction and identify relevant genetic biomarkers for drug-responsiveness in ET.

## Introduction

Essential tremor (ET) is one of the most common movement disorders^1^ affecting around 5% of individuals over 65 years old. The disease causes an 8–12 Hz kinetic tremor that typically affects the upper limbs but can also affect the head, voice, and rarely the lower limbs. Tremor intensity can sometimes increase with age and have a severe impact on activities of daily living. Recent studies aimed at identifying common and rare genetic variants have yielded mixed results, possibly due to clinical heterogeneity thus decreasing power of genetic studies^2^. Only a handful of variants have been identified and even fewer of them were replicated in other studies. Therefore, new approaches are needed, and transcriptomics might yield new insights in the pathophysiology of ET.

Recent studies in psychiatric genetics have successfully used drug effect screens to identify putative disease genes^3,4^. This approach is particularly relevant to diseases that have specific drug-responsive subsets of patients, as is the case with lithium responsive patients in bipolar disorder (BD)^5^. This kind of approach has yet to be used in many drug-responsive neurological disorders such as ET where patients respond to two drugs: propranolol and primidone^6^.

Propranolol and primidone are the most common drug treatments for ET, although both were not initially developed to specifically treat ET. Both are efficient at reducing tremor by about 50% in ET patients^6^. Drug response is variable between patients, with some having a better outcome with either propranolol or primidone. The tremor-reducing effects of propranolol are thought to be due to its dual effect on both muscle spindles in the periphery and CNS effects^7^. Propranolol reduces the excitability of muscles spindles but its effects on neurons in the CNS have yet to be investigated^8^. Evidence demonstrates however that it may act through modifying transcription as shown by its upregulation of *SHF* transcripts, a gene that was shown to be downregulated in the cerebellum of ET patients^9^. The tremor reducing effects of primidone are thought to be mediated through cation channels in the CNS, but the exact neurons or cells through which this effect is mediated are unknown^8,10^. Therefore, the effects of both drugs on CNS cells have yet to be elucidated.

Studying the effects of tremor-reducing drugs on transcription can inform us on mechanisms that reduce tremors. Furthermore, it is possible that genes that are targeted by both drugs are implicated in ET pathophysiology and could allow for the identification of genes harboring putative ET causing variants. Currently, no ET genotypes have been associated with either propranolol or primidone response. Moreover, few ET rare variants have been validated in multiple cohorts^2^. Therefore, since no robust genetic models for ET exist, we set out to study ET drug responses in wild-type cells that are representative of two brain regions associated with ET: the cortex and cerebellum^11^. Even if these drugs have been repurposed to treat ET and are not specific to the disease, understanding their mechanisms in cells from disease-relevant brain regions might yield valuable information on disease pathophysiology itself.

In this study, we identified convergent transcriptomic targets of primidone and propranolol in cortical neural progenitor cells (NPC) and cerebellar medulloblastoma cells (DAOY). Common cellular pathways affected by both treatments were related to neuronal morphology, axon guidance as well as cell-cell interactions as revealed by co-expression and pathway enrichment analysis. We also found that ET drugs specifically affected the expression of genes intolerant to loss-of-function (LoF) variants, hinting at possible enrichment of such rare LoF variants in ET. Furthermore, with integration of single-cell data, we find that drug-targeted genes are mostly enriched in non-neuronal cell types such as endocytes, astrocytes, and oligodendrocytes in both cortical and cerebellar tissues. Our study identifies new putative ET- and tremor-related genes and informs on the molecular and cellular basis for tremor reduction in ET.

## Results

### Differential expression following propranolol and primidone treatment

**Fig. 1:**
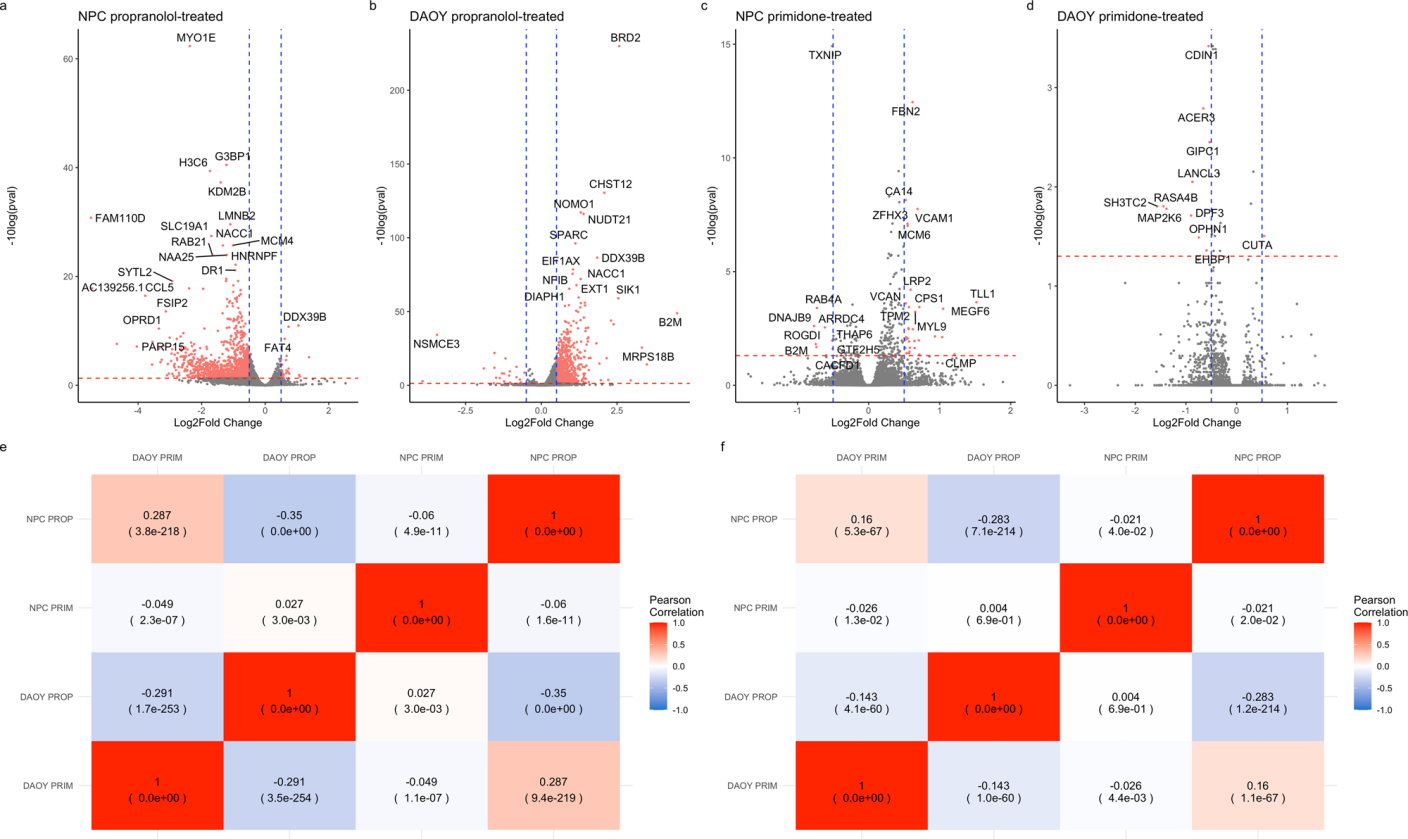
Correlation between DAOYs and NPCs treated with propranolol and primidone. Volcano plots of propranolol-treated NPCs **a** and DAOYs **b** as well as primidone-treated NPCs **c** and DAOYs **d**. Blue lines indicate −0.5- and 0.5-Log2FC changes. Red lines indicate *q* value significance threshold (0.05). **e** Unweighted Pearson correlations between DEGs *z*-scores from different conditions of treatment and cell types. **f** Weighted Pearson correlations between DEGs *z*-scores from different conditions of treatment and cell types.

To assess the transcriptomic effect of propranolol and primidone on neuronal and cerebellar cells, NPCs and DAOYs were independently treated with clinically relevant concentrations of both drugs for 5 days. Differential expression was done using the Wald test (WT) in the R package Sleuth^12^ (Eq. (1)). Treatment of NPCs with propranolol resulted in 1754 DE genes (Supplementary Table 1) while treatment of NPCs resulted in 1571 DE genes (Supplementary Table 2). Directionality of overall transcriptional effect was widely different between NPCs and DAOYs, with propranolol treatment resulting in mostly underexpression in NPCs and overexpression in DAOYs (Fig. 1a, b). Pearson correlation of propranolol-treated NPCs and DAOYs effectively show a weak negative correlation, indicating transcriptomic effects on different genes (*r* = −0.35, *p* val < 2.2E−308, Fig. 1e). This correlation weakens when weighing for the most significant DEGs (*r* = −0.283, *p* = 7.1E−214, Fig. 1f). Primidone, on the other hand, had a weak effect on transcription in both DAOYs and NPCs with only 200 DEGs (Supplementary Table 3) and 23 DEGs (Supplementary Table 4) in each, respectively (Fig. 1c, d for volcano plots). In NPCs, propranolol and primidone DEGs were lowly correlated (*r* = −0.06, *p* val = 1.6E−11, Fig. 1e) with a weaker weighted correlation (*r* = −0.021, *p* val = 2.2E−02, Fig. 1f). Similar weak (weighted and unweighted) correlations are seen between the two drugs in DAOYs (Fig. 1e, f).

### ET drug targets converge on genes related to movement disorders and ET

Shared effects of propranolol and primidone on specific genes increases the likelihood of these genes being integral to tremor reduction in ET. Therefore, convergence of drug effects on expression was assessed by comparing gene *Z*-scores from different treatment conditions: convergent drug targets in either DAOYs or NPCs, convergent propranolol or primidone targets in both cell types and convergent targets of both drugs in all cell types. Table 1 shows the top 35 convergent DEGs across all cells and treatment conditions (see Supplementary Table 5 for full statistics).

**Table 1:** Top 35 convergent DEGs across all conditions.

Gene	Daoy PRIM Log2FC	Daoy Prim *q* val	Daoy Prop Log2FC	DAOY PROP *q* val	NPC PRIM Log2FC	NPC PRIM *q* val	NPC PROP Log2FC	NPC PROP *q* val	Stouffer’s *Z* (convergence)	Stouffer *q* val
*BRD2*	−1.533	9.998E−01	2.567	1.174E−230	−0.043	9.947E−01	−1.238	4.090E−02	14.178	1.430E−41
*CHST12*	−1.536	9.998E−01	2.076	2.961E−131	0.157	9.769E−01	−0.153	7.611E−01	11.536	4.910E−27
*NOMO1*	−0.834	9.998E−01	1.302	7.364E−118	−0.036	9.916E−01	−0.921	6.305E−05	8.488	7.950E−14
*NFIB*	−0.689	9.998E−01	1.031	2.763E−76	0.234	8.052E−01	−0.683	2.418E−01	8.352	1.910E−13
*SPARC*	−0.421	9.998E−01	1.125	6.794E−97	−0.094	5.354E−01	−0.232	1.258E−01	7.826	1.150E−11
*SKI*	−0.870	9.998E−01	1.440	7.651E−55	−0.107	8.877E−01	0.234	2.062E−01	7.780	1.380E−11
*ZMIZ1*	−0.575	9.998E−01	0.939	4.747E−31	0.266	5.758E−02	0.229	6.006E−01	7.590	5.180E−11
*NUDT21*	−0.899	9.998E−01	1.393	7.608E−117	0.036	9.819E−01	−0.887	9.690E−13	7.364	2.530E−10
*TIMM13*	−0.912	9.998E−01	1.303	2.058E−56	0.000	9.992E−01	−0.055	8.565E−01	7.118	1.250E−09
*MYO1E*	−1.328	9.998E−01	0.625	1.515E−01	0.244	3.649E−01	−2.375	4.660E−63	−7.125	1.250E−09
*NONO*	−0.090	9.998E−01	1.230	3.054E−60	−0.050	9.900E−01	−0.089	6.281E−01	6.929	4.370E−09
*USP6NL*	0.043	9.998E−01	0.737	1.463E−26	0.036	9.769E−01	0.105	6.006E−01	6.694	2.060E−08
*B2M*	−3.286	9.998E−01	4.482	1.548E−49	0.509	9.947E−01	−0.298	7.047E−01	6.601	3.580E−08
*ARID1B*	−0.647	9.998E−01	0.813	1.325E−43	0.143	4.174E−01	−0.271	1.746E−01	6.452	8.940E−08
*FAT4*	−0.127	9.998E−01	0.282	6.152E−01	0.351	1.695E−05	0.612	2.871E−09	6.376	1.380E−07
*ARHGAP31*	−0.046	9.998E−01	0.852	3.319E−35	0.147	9.918E−01	−0.299	8.664E−01	6.347	1.560E−07
*GABRG2*	−0.907	2.539E−01	−0.631	2.479E−01	−0.423	9.918E−01	−2.576	2.646E−10	−6.300	1.990E−07
*G3BP1*	−0.782	9.998E−01	0.672	5.807E−01	0.090	6.556E−01	−1.223	3.204E−41	−6.285	2.070E−07
*BRD3*	0.109	9.998E−01	0.937	2.216E−24	0.128	9.739E−01	0.013	9.746E−01	6.117	5.720E−07
*ATF6B*	−0.962	9.998E−01	2.386	4.626E−42	0.056	9.842E−01	−0.188	2.577E−01	6.088	6.510E−07
*TRIM44*	−0.520	9.998E−01	0.820	1.726E−36	0.044	9.947E−01	−0.011	9.777E−01	6.064	7.180E−07
*AP2M1*	−0.343	9.998E−01	0.782	3.849E−44	0.091	9.939E−01	−0.212	2.840E−01	5.947	1.410E−06
*UTP4*	−1.240	9.983E−01	2.301	1.442E−44	−0.215	7.209E−01	0.115	7.622E−01	5.910	1.690E−06
*ROBO1*	−0.114	9.998E−01	0.358	3.798E−05	0.326	2.230E−04	0.253	5.121E−02	5.866	2.120E−06
*TNRC6B*	−0.243	5.337E−01	0.844	1.741E−26	0.164	6.018E−01	0.165	4.706E−01	5.845	2.310E−06
*MAP1B*	−0.043	9.998E−01	0.105	3.132E−01	0.289	5.008E−09	0.273	9.399E−03	5.787	3.140E−06
*KDM2B*	−0.335	9.998E−01	0.081	7.684E−01	0.137	5.815E−01	−1.401	5.299E−38	−5.757	3.600E−06
*PDZD8*	−0.392	9.998E−01	0.839	5.705E−27	0.081	9.564E−01	0.100	7.470E−01	5.739	3.860E−06
*WTIP*	−0.031	9.998E−01	2.154	4.783E−19	0.203	8.879E−01	0.653	6.115E−01	5.724	4.070E−06
*ACOT13*	−0.556	9.998E−01	1.288	1.402E−53	−0.089	9.784E−01	−0.913	3.681E−02	5.699	4.420E−06
*FSIP2*	−1.581	7.308E−01	−0.171	6.495E−01	0.463	9.936E−01	−3.129	2.668E−14	−5.704	4.420E−06
*CHD7*	−0.319	9.998E−01	1.263	4.737E−23	0.380	3.162E−01	−0.054	8.085E−01	5.664	5.270E−06
*IRX3*	−0.148	9.998E−01	1.292	7.149E−23	−0.214	9.090E−01	0.368	1.240E−01	5.612	6.880E−06
*SLC45A4*	−0.481	9.998E−01	1.195	9.556E−25	0.147	8.415E−01	0.028	9.455E−01	5.542	9.980E−06
*PLAA*	−0.198	9.998E−01	0.692	3.441E−25	0.302	9.325E−01	−0.015	9.698E−01	5.535	1.010E−05

Across DAOYs and NPCs, 788 significant convergent DEGs were found with propranolol treatment (Supplementary Table 6), 36 convergent DEGs following primidone treatment (Supplementary Table 7) and 265 convergent DEGs across all conditions (Table 1 and Supplementary table 5 for full list). In total, 210 propranolol and 12 primidone-specific convergent DEGs were also found to be convergent across both treatments (e.g., *BRD2, MYO1E, ROBO1*, etc; Table 1) Propranolol increased expression of *TRAPPC11*, a trafficking protein previously associated with ET^9^, in DAOYs (log2FC = 0.98, *q* val = 5.32E−27) and this gene was also found to be convergently affected across both cell lines (Stouffer’s *Z*-score = 5.41, *q* val = 5.87E−06; Supplementary Table 6). Propranolol also decreased expression of *G3BP1* in NPCs (Log2FC = −1.22, *q* val = 3.20E−41) which encodes a protein implicated in stress granule formation and is known to affect axonal mRNA translation as well as nerve regeneration^13^. This effect was also found to be convergent across both NPCs and DAOYs (Stouffer’s *Z*-score = −9.07, *q* val = 7.84E−17). *BRD2*, a transcription factor previously implicated with epilepsy, was convergently upregulated following propranolol treatment in both cells (DAOY Log2FC = 2.57, *q* val = 1.17E−230; NPC Log2FC = 0.360, *q* val = 0.007; Stouffer’s *Z*-score = 21.13, *q* val = 4.56E−95). *NONO,* a gene harboring a splicing variant known to cause X-linked intellectual deficiency with intentional tremor, was found to be upregulated in DAOYs treated with propranolol (Log2FC = 1.23, *q* val = 3.05E−60)^14^. Primidone, in NPCs, upregulated *VCAM1* (Log2FC = 0.69, *q* val = 1.77E−08) and this effect was found to be convergent across both cell lines *(*Stouffer’s *Z*-score = 5.53, *q* value = 1.29E−04). *VCAM1* is a gene implicated in axonal myelination by oligodendrocytes^15^. *GIPC1* was also found to be convergently downregulated following primidone treatment when leveraging effects in both cell types (DAOY Log2FC = −0.534, *q* val = 0.004; NPC Log2FC = −0.360, *q* val = 0.137; Stouffer’s *Z*-score = −5.46, *q* val = 1.42E−04). GIPC1 is a known interactor of DRD3 which has previously been associated with ET and Parkinson’s (PD)^2,16,17^.

### Propranolol and primidone act on pathways related to neuronal survival as well as axon guidance

Following the identification of convergent DEGs across treatments, we aimed to identify molecular pathways affected by propranolol and primidone in DAOYs and NPCs. Co-expression enrichment analysis (using GeneNetwork2.0^18^) for convergent DEGs across all conditions showed that Reactome terms related to GPCR signaling (*q* val = 1.12E−19), axon guidance (*q* val = 1.68E−08), Semaphorin interactions (*q* val = 3.24E−13) and VEGF signaling (*q* val = 2.23E−08) were significantly enriched within the convergent genesets (Supplementary Table 14). Furthermore, Ca^2+^ signaling (*q* val = 4.67E−07) and voltage-gated potassium channels (*q* val = 4.64E−06) were also found to be significantly enriched. Interestingly, GO:cellular components significant terms were mostly related to cell:cell or cell:extracellular matrix interactions as well as axon guidance such as lamellipodium (*q* val = 4.47E−13), filopodium (*q* val = 3.54E−11), focal adhesion (*q* val = 4.70E−11) and growth cone (*q* val = 1.04E−09)(Supplementary Table 16).

Pathway enrichment analysis of convergent propranolol DEGs (in both cell types) was also performed using g:profileR using genes expressed in both DAOYs and NPCs as background (Table 2). Pathways known to be affected by propranolol such as HIF-1alpha (*q* val = 0.001) and regulation of apoptosis (*q* val = 0.02) were significantly enriched. Much like the co-expression analysis, Reactome terms related to axon guidance were found to be significant, such as RUNX1 transcription (*q* val = 0.0002), a transcription factor implicated in growth cone guidance of DRG neurons^19^. Interestingly, CaMKK2 signaling pathway was found to be significantly enriched within genes in the propranolol geneset. *CAMKK2* encodes a kinase implicated in synapse homeostasis and is also involved in modifying Aβ synaptotoxicity in Alzheimer’s disease^20^.

**Table 2:** Pathway enrichment for convergent propranolol DEGs in both DAOYs and NPCs.

Source	Term	*Q* value
CORUM	PA700 complex	0.007
CORUM	p54(nrb)-PSF-matrin3 complex	0.007
CORUM	PA700-20S-PA28 complex	0.013
CORUM	HEXIM1-DNA-PK-paraspeckle components-ribonucleoprotein complex	0.051
CORUM	Ubiquitin E3 ligase (CHEK1, CUL4A)	0.065
CORUM	CORUM root	0.077
CORUM	EBAFb complex	0.089
CORUM	NCOR1 complex	0.089
KEGG	Proteasome	0.009
KEGG	Spinocerebellar ataxia	0.027
KEGG	Prion disease	0.047
KEGG	Protein processing in endoplasmic reticulum	0.053
KEGG	Hippo signaling pathway—multiple species	0.090
MIRNA	hsa-miR-6766-5p	4.37E-04
MIRNA	hsa-miR-6756-5p	4.37E-04
MIRNA	hsa-miR-539-5p	4.39E-04
MIRNA	hsa-miR-4668-3p	0.007
MIRNA	hsa-miR-21-5p	0.013
MIRNA	hsa-miR-654-5p	0.021
MIRNA	hsa-miR-541-3p	0.027
MIRNA	hsa-miR-1468-3p	0.044
MIRNA	hsa-let-7b-5p	0.046
MIRNA	hsa-miR-548f-5p	0.051
MIRNA	hsa-miR-548aj-5p	0.055
MIRNA	hsa-miR-548x-5p	0.055
MIRNA	hsa-miR-548g-5p	0.055
MIRNA	hsa-miR-193b-3p	0.055
REAC	Transcriptional regulation by RUNX1	2.26E-04
REAC	Oxygen-dependent proline hydroxylation of Hypoxia-inducible Factor Alpha	0.001
REAC	Cellular response to hypoxia	0.004
REAC	Host Interactions of HIV factors	0.004
REAC	Cell Cycle Checkpoints	0.007
REAC	UCH proteinases	0.007
REAC	G2/M Checkpoints	0.012
REAC	Regulation of ornithine decarboxylase (ODC)	0.012
REAC	G1/S DNA Damage Checkpoints	0.013
REAC	Signaling by NOTCH	0.014
REAC	p53-Independent G1/S DNA damage checkpoint	0.015
REAC	Ubiquitin Mediated Degradation of Phosphorylated Cdc25A	0.015
REAC	p53-Independent DNA Damage Response	0.015
REAC	Regulation of APC/C activators between G1/S and early anaphase	0.015
REAC	Regulation of Apoptosis	0.017
REAC	Cdc20:Phospho-APC/C mediated degradation of Cyclin A	0.021
REAC	Assembly of the pre-replicative complex	0.023
REAC	Deubiquitination	0.024
REAC	Autodegradation of Cdh1 by Cdh1:APC/C	0.024
REAC	APC:Cdc20 mediated degradation of cell cycle proteins prior to satisfaction of the cell cycle checkpoint	0.025
REAC	Regulation of MECP2 expression and activity	0.029
REAC	Stabilization of p53	0.031
REAC	APC/C:Cdc20 mediated degradation of mitotic proteins	0.033
REAC	DNA Replication Pre-Initiation	0.033
REAC	Orc1 removal from chromatin	0.034
REAC	PTEN Regulation	0.034
REAC	Metabolism of polyamines	0.036
REAC	Activation of APC/C and APC/C:Cdc20 mediated degradation of mitotic proteins	0.038
REAC	Regulation of mitotic cell cycle	0.040
REAC	APC/C-mediated degradation of cell cycle proteins	0.040
REAC	Transcriptional regulation by RUNX3	0.040
REAC	CDT1 association with the CDC6:ORC:origin complex	0.041
REAC	MAPK6/MAPK4 signaling	0.042
REAC	Ub-specific processing proteases	0.043
REAC	Switching of origins to a post-replicative state	0.043
REAC	APC/C:Cdc20 mediated degradation of Securin	0.045
REAC	Vpu mediated degradation of CD4	0.054
REAC	Cross-presentation of soluble exogenous antigens (endosomes)	0.072
REAC	Regulation of activated PAK-2p34 by proteasome mediated degradation	0.072
REAC	Hedgehog ligand biogenesis	0.074
REAC	p53-Dependent G1/S DNA damage checkpoint	0.082
REAC	p53-Dependent G1 DNA Damage Response	0.082
REAC	SCF-beta-TrCP mediated degradation of Emi1	0.087
REAC	CDK-mediated phosphorylation and removal of Cdc6	0.091
REAC	Autodegradation of the E3 ubiquitin ligase COP1	0.095
REAC	Ubiquitin-dependent degradation of Cyclin D	0.095
WP	mRNA Processing	0.004
WP	CAMKK2 Pathway	0.004
WP	Pathways Affected in Adenoid Cystic Carcinoma	0.017
WP	MET in type 1 papillary renal cell carcinoma	0.024
WP	Oncostatin M Signaling Pathway	0.078
WP	15q13.3 copy number variation syndrome	0.080
WP	Gastrin Signaling Pathway	0.090

**Fig. 2:**
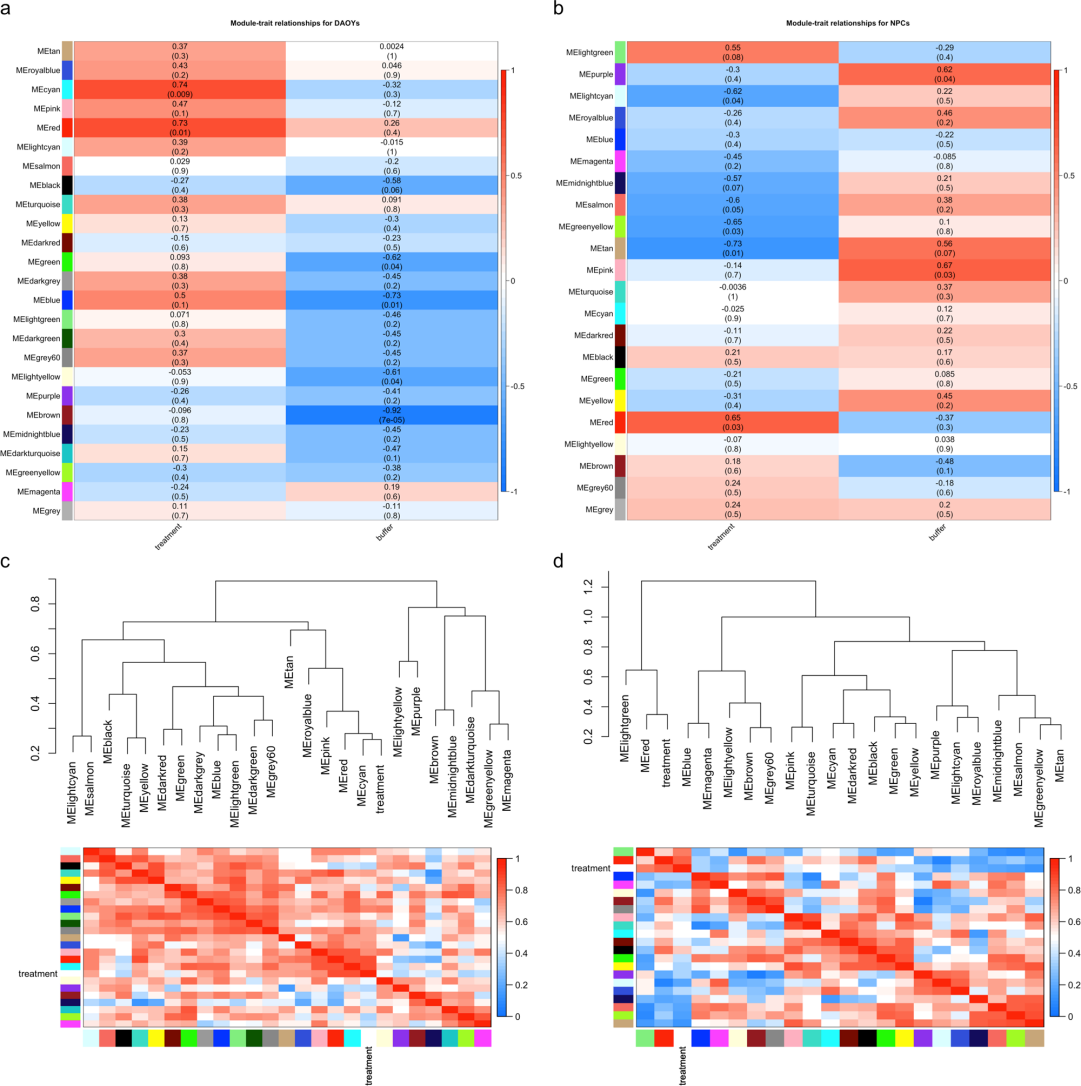
Co-expression gene modules for convergent propranolol and primidone targets. **a** Module-treatment (propranolol/primidone) and -buffer (H2O/DMSO; control) correlation heatmaps for DAOYs. **b** Module-treatment (propranolol/primidone) and -buffer (H2O/DMSO; control) correlation heatmaps for NPCs. Value indicates correlation between gene-trait and gene-module associations with *p* value in parenthesis. **c** Module dendrograms with module membership correlation heatmaps for DAOYs. **d** Module dendrograms with module membership correlation heatmaps for NPCs.

Weighted gene correlation network analysis was also performed to identify co-expression modules associated with combined propranolol/primidone treatment. Module-trait and module correlation heatmaps are shown in Fig. 2. Two modules (cyan and red; corr = 0.74, *p* val = 0.009; corr = 0.73, *p* val = 0.01 respectively; Fig. 2a) were found to be significantly associated with treatment in DAOYs and only one module (red; corr = 0.65, *p* val = 0.03) was significantly associated with NPCs (Fig. 2b). Pathway enrichment analysis of DAOY red module genes found an enrichment of Reactome terms related to RABGAP signaling (*q* val = 0.009) as well as RUNX1 transcription (*q* val = 0.02; Table 3). NPC red modules genes were significantly associated with neuronal morphology, axon guidance and neurogenesis (Table 4).

**Table 3:** Pathway enrichment analysis of red gene module for drug treatment in DAOYs.

Source	Term	*Q* value
CORUM	Ubiquitin E3 ligase (CCDC22, COMMD8, CUL3)	0.005
CORUM	Ecsit complex (ECSIT, MT-CO2, GAPDH, TRAF6, NDUFAF1)	0.074
REAC	TBC/RABGAPs	0.010
REAC	RUNX3 regulates YAP1-mediated transcription	0.023
REAC	RNA polymerase II transcribes snRNA genes	0.0856
REAC	Rab regulation of trafficking	0.093
WP	Eukaryotic Transcription Initiation	0.091

**Table 4:** Pathway enrichment analysis of red gene module for drug treatment in NPCs.

Source	Term	*Q* value
CORUM	AML1-HIPK2-p300 complex	0.017
CORUM	EGR-EP300 complex	0.023
CORUM	DNA polymerase alpha-primase complex	0.042
CORUM	TNF-alpha/NF-kappa B signaling complex 9	0.043
GO:BP	cell morphogenesis	9.93E−09
GO:BP	neuron development	4.57E−07
GO:BP	neuron projection development	7.92E−07
GO:BP	cell morphogenesis involved in differentiation	2.46E−06
GO:BP	neuron differentiation	3.78E−06
GO:BP	anatomical structure morphogenesis	5.15E−06
GO:BP	generation of neurons	5.51E−06
GO:BP	neurogenesis	7.43E−06
GO:BP	cell projection morphogenesis	4.04E−05
GO:BP	cellular component morphogenesis	5.32E−05
GO:BP	cell part morphogenesis	8.74E−05
GO:BP	plasma membrane bounded cell projection morphogenesis	1.07E-04
GO:BP	nervous system development	1.19E-04
GO:BP	neuron projection morphogenesis	1.78E-04
GO:BP	cell morphogenesis involved in neuron differentiation	3.17E-04
GO:BP	plasma membrane bounded cell projection organization	3.18E-04
GO:BP	cell projection organization	4.36E-04
GO:BP	morphogenesis of an epithelium	8.95E-04
GO:BP	regulation of cell projection organization	0.001
GO:BP	tissue morphogenesis	0.001
GO:BP	regulation of plasma membrane bounded cell projection organization	0.002
GO:BP	regulation of neuron projection development	0.004
GO:BP	axon development	0.004
GO:BP	cell development	0.0067
GO:BP	system development	0.006
GO:BP	positive regulation of cell projection organization	0.023
GO:BP	axonogenesis	0.027
GO:BP	regulation of anatomical structure morphogenesis	0.033
GO:BP	developmental growth	0.040
MIRNA	hsa-miR-218-5p	0.002
REAC	Nervous system development	0.013
REAC	Axon guidance	0.033
REAC	Attenuation phase	0.049
WP	Pathways Affected in Adenoid Cystic Carcinoma	2.59E-04
WP	Mesodermal Commitment Pathway	0.028

### Correlation of the effects of propranolol and primidone with those of common and rare variants in ET

**Fig. 3:**
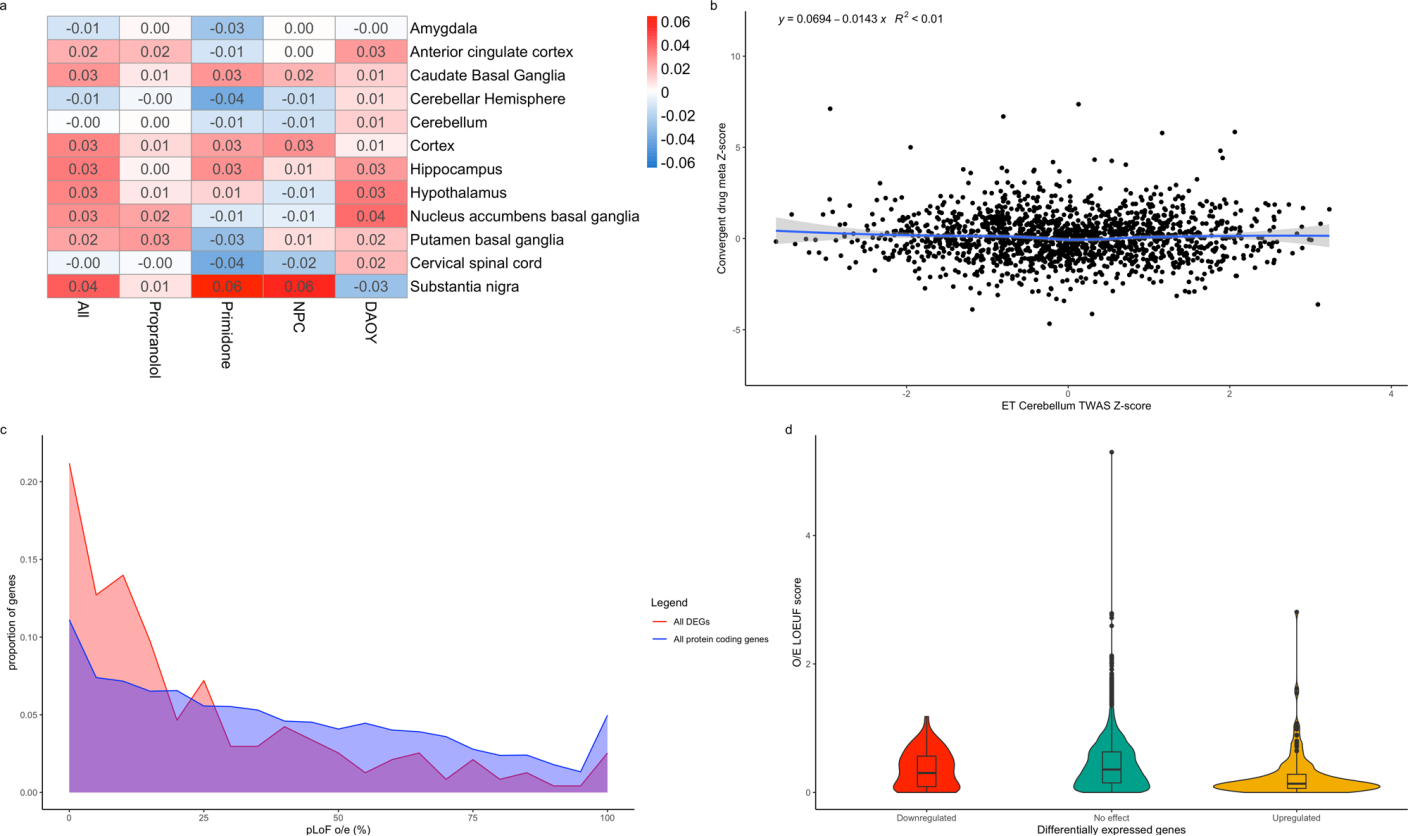
Effects of ET drugs on common and rare variants. **a** Correlation heatmap of ET TWAS gene *Z*-scores in different brain tissues and drug effect gene *Z*-scores from different meta-analysis conditions. Values indicate *Z*-score regression coefficient from linear model. **b** Correlation plot of TWAS gene *Z*-scores from cerebellar tissue and convergent primidone and propranolol gene *Z*-scores across DAOYs and NPCs. **c** Line histogram displaying the distribution of O/E LOEUF scores from all protein coding genes (blue) and convergent DEGs (red) following drug treatment. O/E scores were directly transformed to percentages (ex. 0.25 as 25%) with scores over 10 counted as 100%. **d** Violin plots of O/E LOEUF scores for upregulated DEGs (yellow), downregulated DEGs (red) and non-significant DEGs (green). Boxplot elements: center line = median; upper and lower hinges = 1st and 3rd quartiles respectively; whiskers = mean ± interquartile range × 1.5.

TWAS studies the effect of common SNPs associated with a disease on the expression of genes in different tissues. We postulated that transcriptomic targets of propranolol and primidone might correlate with the transcriptomic effect of common ET variants. We used TWAS summary statistic from the latest ET GWAS^21^ to measure the correlation between TWAS gene Z-scores and convergent drug target *Z*-scores (across all possible conditions) while controlling for gene length and GC content (Eqs. (2) and (3)). Weak, non-significant correlations between TWAS *Z*-scores and drug target *Z*-scores were found across all conditions and all brain tissues (*p* > 0.05; Fig. 3a). Cerebellar hemispheres and cerebellum tissues, brain regions highly associated with ET, displayed no correlations with convergent drug targets (coeff = −0.0143, *p* val = 0.549; coeff = −0.000138, *p* val = 0.994 respectively; Fig. 3b).

We postulated that since propranolol and primidone had a non-significant correlation with expression of genes harboring common variants for ET, they might instead act on genes that have rare variants. GnomAD recently published observed/expected (o/e) LoF scores for all protein coding genes in the genome. These scores inform on the tolerance of genes to rare LoF variants, with genes with a higher frequency of observed to expected LoF variants being more tolerant to mutations. Figure 3c shows the distribution of LoF scores of drug DEGs compared to all protein coding genes passing the initial DE QC. Drug targets displayed a significantly lower o/e score median (*n* = 256, median = 0.18) than all protein coding genes (*n* = 11,188, median = 0.36; *W* = 1,727,520, *p* val = 1.50E−10) using a Wilcoxon unpaired test. Interestingly, when looking at Log2FC direction (Fig. 3d), upregulated genes (*n* = 194) had a significantly lower o/e score median (median = 0.15, *W* = 1,361,482, *p* val = 2.917E−12) than all protein coding genes whilst no significant difference was found between o/e scores medians of downregulated genes (*n* = 71) and all protein coding genes (median = 0.35, *W* = 417,126, *p* value = 0.3246) using a Wilcoxon unpaired test. Thus, propranolol and primidone increased expression of mutationally constrained genes in cultured DAOYs and NPCs.

### Single-cell enrichment of propranolol and primidone-targeted genes

**Fig. 4:**
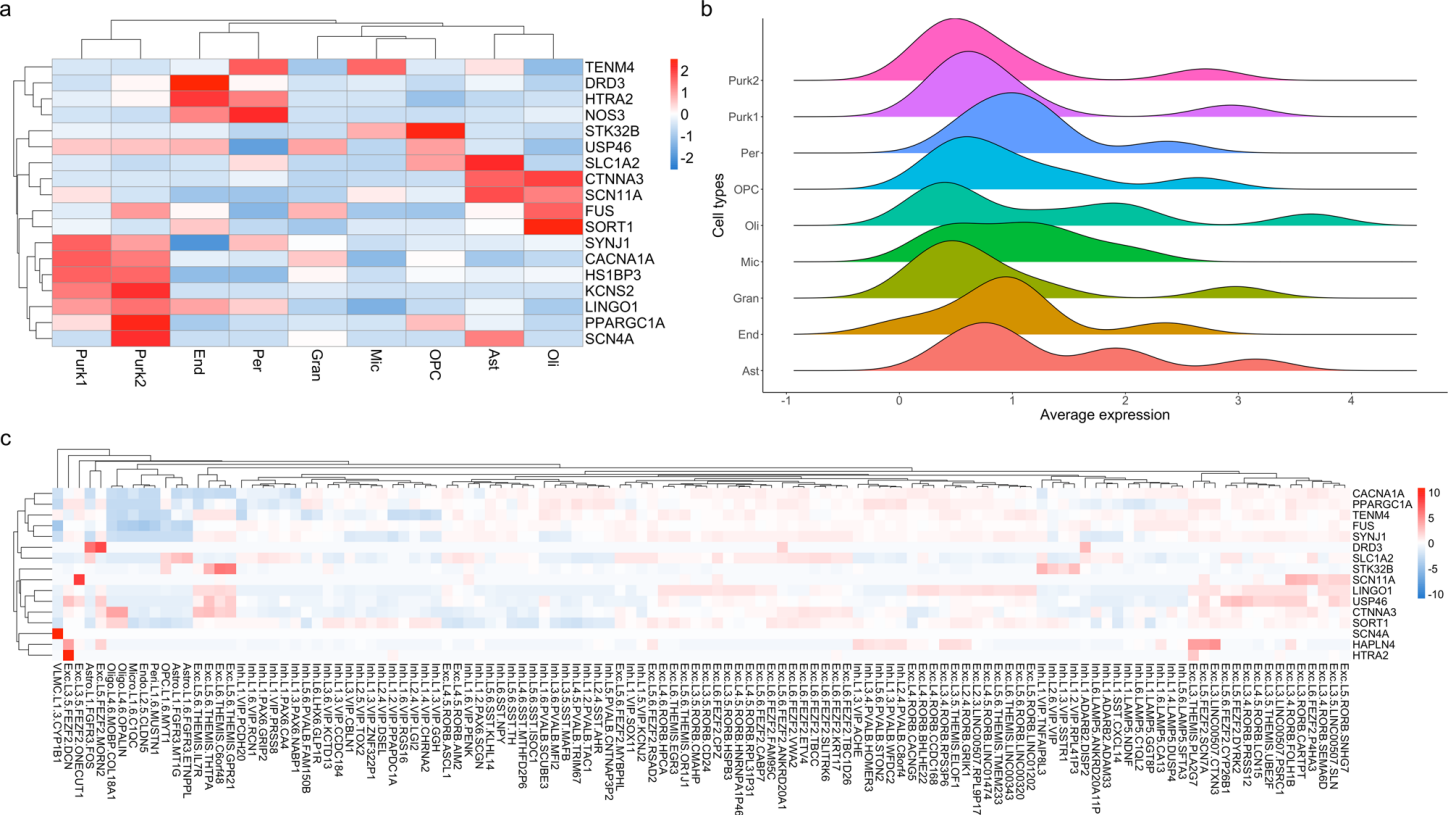
Single-cell enrichment of ET genes in cerebellar and cortical tissues. **a** Single-cell enrichment *Z*-score heatmap of ET-related genes in adult cerebellar tissue. Rows represent ET genes; columns represent cell types of the cerebellum (Purk1 SORC3+ Purkinje cells, Purk2 SORC3− Purkinje cells, Ast Astrocytes, OPC Oligodendrocyte progenitor cells, Oli Oligodendrocytes, Mic Microglia, End Endocytes, Gran Granule cells, Per Pericytes). **b** Ridge plots displaying distribution of average expression counts of ET-related genes in different cell types of the adult cerebellum. **c** *Z*-score expression heatmap of ET genes in single-cell types of the adult cortex. Rows represent ET genes; columns represent cortical cell types (Exc Excitatory, Inh Inhibitory, Astro Astrocytes, End Endocytes, Peri Pericytes, VLMC vascular and leptomeningeal cells, OPC Oligodencrocyte progenitor cells, Oligo Oligodendrocytes, L# cortical layer).

**Fig. 5:**
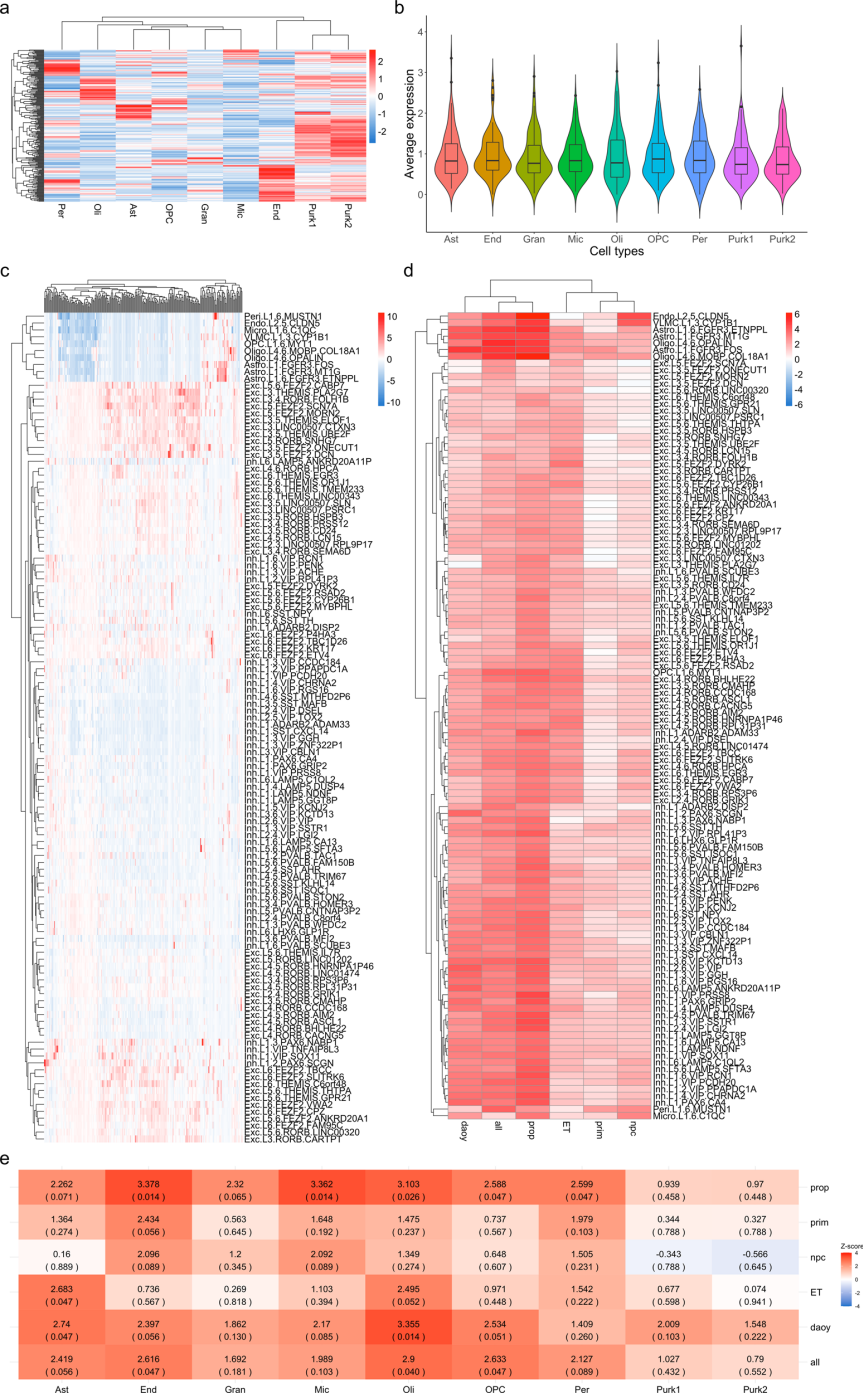
Single-cell enrichment of drug DEGs in cerebellar and cortical tissues. **a** Single-cell enrichment *Z*-score heatmap of convergent propranolol/primidone DEGs in adult cerebellar tissue. Rows represent DEGs; columns indicate cell types; legend color scheme is based on enrichment *z*-score direction. **b** Violin plot of average expression per cerebellar cell type of convergent propranolol/primidone DEGs. Boxplot elements: center line = median; upper and lower hinges = 1st and 3rd quartiles respectively; whiskers = mean ± interquartile range × 1.5. **c** Single-cell enrichment *Z*-score heatmap of convergent propranolol/primidone DEGs in adult cortical tissue. Rows represent cell types; columns indicate DEGs; legend color scheme is based on enrichment *Z*-score direction. **d** Enrichment *Z*-score heatmap of DEGs gene-sets from different conditions (see below for abbreviations) in single-cell data from adult cortex. Rows represent cell types; columns represent condition gene-sets. **e** Enrichment *Z*-score heatmap of DEGs gene-sets from different conditions in single-nucleus sequencing data from adult cerebellar tissue. Rows indicate condition gene-sets; columns indicate cerebellar cell types. ET ET-related genes, prop convergent propranolol DEGs in both cell types, prim convergent primidone DEGs in both cell types, DAOY convergent propranolol and primidone DEGs in DAOY cells only, NPC convergent propranolol and primidone DEGs in NPCs only, all convergent propranolol and primidone DEGs in both cell type.

Our current understanding of CNS cell types affected in ET is still very limited. Enrichment of disease related genes can indirectly inform on potential cell types implicated in disease pathophysiology^22^. We first sought to assess the enrichment of ET genes discovered through familial linkage, whole-exome, GWAS and transcriptomic studies in cell types of the adult cerebellum and cerebral cortex (Figs. 4 and 5 and Supplementary Tables 17–18). Enrichment *Z*-scores per cell type for ET genes as well as drug DEGs were calculated based on average normalized expression in single-nucleus cerebellum data from Lake et al.^23^ and cortical single-cell Smart-seq data from the Allen Brain Institute. In the cerebellum, ET genes were mostly enriched in astrocytes (enrichment *z*-score = 3.11, *q* value = 0.021; Fig. 4a, b). In the cortex, the strongest enrichments of ET genes were found in oligodendrocyte progenitor cells (OPCs; *z*-score = 3.55) and L3-L5 excitatory neurons with the most significant neuronal cell type being the *FEZF2-, DYRK*-expressing pyramidal neurons of cortical layer V (*z*-score = 3.28, *q* val = 0.0068; Fig. 4c). Significant enrichment was also found in L1 *MTG1* astrocytes (*z*-score = 3.13, *q* val = 0.0090).

Next, we assessed the enrichment of propranolol and primidone DEGs identified in this study in cortical and cerebellar single-cell data using a one sample *Z*-test (Eq. (4); Fig. 5 and Supplementary Tables 17 and 18). In cerebellum single-nucleus data, convergent propranolol DEGs were mostly enriched in endocytes (*z*-score = 3.38, *q* val = 0.014) and microglia (*z*-score = 3.36, *q* val = 0.014) whilst convergent propranolol/primidone DEGs in all cell types were mostly enriched in oligodendrocytes (*z*-score = 2.90, *q* val = 0.034; Fig. 5e). Interestingly, convergent propranolol/primidone DEGs in DAOYs, a cell type specific to the cerebellum, had enriched expression in astrocytes (*z*-score = 2.74, *q* val = 0.047), much like the enrichment of ET genes in cerebellar astrocytes (Fig. 4a). In cortical tissue, convergent drug DEGs were mostly significantly enriched in non-neuronal cell types (Fig. 5d), notably oligodendrocytes (*z*-score = 5.09, *q* val = 3.65E−07), astrocytes (*z*-score = 4.92, *q* val = 1.00E−04) and endocytes (*z*-score = 3.95, *q* val = 1.70E−03). Unsurprisingly, given the use of propranolol to lower blood pressure, convergent propranolol DEGs were mostly enriched in endocytes (*z*-score = 6.18, *q* val = 4.48−07) and vascular and leptomeningeal cells (*z*-score = 4.77, *q* val = 1.52E−04). Of note, propranolol DEGs were also enriched in L1-L3 inhibitory neurons, notably vasoactive intestinal peptide (VIP) expressing inhibitory neurons (Fig. 5d and see Supplementary Tables 17 and 18 for statistics).

## Discussion

Understanding the cellular and molecular mechanisms behind drug treatments can inform on disease pathophysiology. In this study, we sought to investigate the transcriptomic effects of first line treatments for ET in cerebellar DAOY cells as well as NPCs, to gain insight on potential disease related genes. Overall, weak Pearson correlations were observed between the same treatment in different cells indicating that the drugs might have completely different effects on genes and pathways in the cortex compared to the cerebellum. Nonetheless, 265 genes were found to be convergent across both treatment and cell types. Indeed, we found that propranolol and primidone affected expression of multiple genes related to movement disorders and ET. Notably, *TRAPPC11*, whose expression was previously shown to be altered in ET cerebellar cortex and is also involved in protein trafficking^9^. Other genes related to endosomal trafficking were found to be differentially expressed after propranolol treatment, such as *MYO1E* and *SYNJ1*. Convergent DEGs also displayed an enrichment of genes related to the ESCRT complex, known to be a pillar of endosomal trafficking in neurons. These findings potentially increase the likelihood of endosomal trafficking being altered in ET and possibly partly restored through transcriptomic effects of propranolol.

Axon guidance was previously associated with ET in several studies^2,9,11,24,25^. Bulk-RNA-sequencing of cerebellar cortex and dentate nucleus of ET patients showed a significant enrichment of axon guidance genes^9^. Hallmark axon guidance genes such as *ROBO1* (*z*-score = 5.87, *q* val = 1.88E−06) and *NEO1* (*z*-score = 4.01, *q* val = 5.04E−03) were both found to have increased expression following drug treatment. NEO1 (and its paralog DCC), which binds netrin-1, is implicated in cell-cell adhesions, mostly between axons and oligodendrocytes, as well as cell-extracellular matrix adhesions. Netrin-1 also acts on dendrite arborization, increasing connections in excitatory synapses^26^. Interestingly, NEO1 protein remains expressed in Purkinje cells of the adult cerebellum (GTEx V8). Thus, the post-developmental role of axon guidance signaling pathways is to maintain adhesions and important synaptic connections between cells. This might be an important process by which ET tremorolytic drugs diminish tremor. These findings on axon guidance are concordant with other Reactome/GO-terms found to be enriched amongst DEGs, most notably semaphorin interactions, cadherin binding, and actin cytoskeleton reorganization. Purkinje cell axons in ET patients have shown accumulations of disordered neurofilaments (“axonal torpedoes”) leading to abnormal axonal morphologies^11^. This process is thought to either be part of a neurodegenerative cascade or a response to neurodegeneration. Moreover, decreased neuronal density was observed in multiple brain regions of ET patients, most notably the inferior cerebellar peduncles through which afferent axons from the brainstem nuclei pass in order to reach the cerebellar cortex^27^. Our findings therefore provide additional support for the involvement of axon guidance molecules in ET pathophysiology.

We also identified the CaMKK2 signaling pathway as significantly enriched in propranolol DEGs in DAOYs and NPCs. CaMKK2 exacerbates amyloid-b synaptotoxicity in Alzheimer’s disease through Tau protein phosphorylation by AMPK^20^. This pathway is sensitive to cellular calcium intake, which was shown to be affected at the transcriptome level by both propranolol and primidone. Both Tau protein and amyloid-beta abnormalities have been observed in ET cerebellar tissues, with multiple findings pointing toward protein aggregation being a hallmark of the disease^28,29^. Propranolol affecting transcription of genes implicated in both CAMKK2 and Ca^2+^ signaling pathways might imply that ET drugs could reduce aggregate-induced neurotoxicity.

Convergent drug DEGs did not correlate with transcriptomic effects of common ET variants (TWAS DEGs). Moreover, propranolol and primidone DEGs displayed weak non-significant correlations with gene expression in the cerebellum of ET patients, the principal brain region affected in this disorder^1^. There are several possible explanations for these results. The relatively underpowered state (for a common disease) of the current ET GWAS might not capture the effects of common variation on transcription, in part explaining the absence of correlation with drug DEGs. Moreover, the lack of good cell models for cerebellar neurons as well as the neurodevelopmental state of NPCs also impair adequate comparisons between TWAS statistics and drug DEGs presented in this study.

Convergent drug DEGs are significantly more likely to be genes predicted to be intolerant to LoF variants. Mutationally constrained genes are more likely to be essential for cell homeostasis and survival and thus more likely to be implicated in disease when affected by LoF mutations^30^. Given that both ET drugs converged on these genes in multiple cell types increases the likelihood that these genes harbor rare variants associated with ET. Upregulated DEGs were found to be significantly less tolerant than all protein coding genes while downregulated DEGs were as tolerant as all protein coding genes. These genes could be good candidates for future targeted sequencing, especially within propranolol and primidone responsive cohorts.

Identifying cell types affected in ET remains difficult. Several conflicting studies have tried to identify specific pathological morphologies in post-mortem cerebellum of ET patients, most notably in Purkinje cells, yet no defining histopathological markers have been found^11^. Here we sought to identify the relevant ET cell types by assessing the enrichment of variant-harboring ET genes within single cells in cerebellar and cortical tissues. Expression of ET genes were mostly enriched within L3-L5 excitatory neurons in the cerebral cortex, more specifically *FEZF2* L5 glutamatergic pyramidal neurons^31^. These neurons originate in the primary motor cortex (M1) and form the corticospinal tract that projects to lower motor neurons, which controls conscious movements. These neurons are influenced by multiple cortico-cortical pathways but also input from the cerebellothalamic tract, crucial for movement coordination. The primary motor cortex has previously been shown to be important for tremor generation in ET as subdural stimulation of M1 can reduce tremor intensity in patients^32^. Moreover, propranolol-targeted genes were mostly enriched in VIP-expressing inhibitory neurons of L1-L3. These neurons are known to inhibit motor neurons through different cortical pathways^33^. The enrichment of ET genes within M1 pyramidal neurons coupled with the enrichment of ET drug genes in motor neuron-inhibiting cells does suggest new potential cellular mechanisms through which tremor generation (and/or reduction) occurs in ET.

In the cerebellum, both ET genes and convergent drug DEGs were significantly enriched within astrocytes in the cerebellum. This somewhat contradicts previous histopathological findings postulating that Purkinje cells were the defining cell type in ET pathophysiology. Not much is known about the role of astrocytes in ET but based on other neurodegenerative diseases, it could be argued that they may play an important role in the onset or development of the disease^11^. Oligodendrocytes, whose dysfunction contributes to numerous other neurological diseases, also showed an enrichment of propranolol and primidone-targeted genes. Both astrocytes and oligodendrocytes might be targeted by ET drugs to reduce tremor since non-neuronal cell types are known to be involved in neurodegeneration in numerous diseases^34^. The lack of single-cell data on ET tissues is a limitation in the study of this disease but our results highlight a possible role for non-neuronal cells in the cerebellum in ET.

This study has a number of limitations. Propranolol and primidone are known to act on cell excitability and this effect was postulated as being important for tremor reduction in ET. Given that DAOYs and NPCs are non-excitable, it is very hard to assess the electrophysiological effects of these drugs in these cells. Moreover, the electrophysiological effects of drugs on cells are known to influence transcription^35^. This might explain why primidone had such a mild effect on transcription in both DAOYs and NPCs. The lack of transcriptomic effects of primidone might also be related to the low expression of certain TRP channels by both NPCs and DAOYs which are predicted to be affected by the drug in the context of ET. Contrary to propranolol which acts on GPCRs that mediate multiple effects on gene expression, primidone might convey its tremor reducing actions by short-term effects on cell excitability. These effects would evade our transcriptomic screen performed over the span of multiple days. Moreover, cells used in this study do not represent the complete range of cell types in the cortex and cerebellum. NPCs do not completely replicate neuronal expression and do have a more neurodevelopmental transcriptomic state. DAOYs, on the other hand, are derived from cancerous cells and do have dysregulated expression of genes related to cell division and cell growth. Moreover, these cells do not accurately replicate the disease-state present in cerebellar and cortical neurons and other cells in ET patients. These cells are therefore not specific cellular ET models but are nonetheless helpful in understanding the effects of ET drugs in an ET-related cellular context. This study only serves as an ET drug effect screen and remains a steppingstone for more in-depth functional studies, leveraging better ET models such as patient derived iPSCs.

Our study identifies multiple cellular and molecular pathways implicated in ET pathophysiology and tremor reduction by both propranolol and primidone. Our findings also suggest a role for genes harboring potentially rare, deleterious variants associated with ET. Targeted sequencing of these convergent drug genes in case-control cohorts could help to confirm or infirm this hypothesis. These genes could also be used as biomarkers for propranolol treatment in responsive ET patients. Our results also identify several cell types involved in ET in both cerebellar and cortical tissues, as well as cells potentially affected by propranolol and primidone through which tremor might be reduced. We believe that this relatively novel paradigm to study pharmacogenomics could be leveraged to repurpose other drugs to treat ET. Moreover, this approach could be used in other diseases to understand the biological effects of drugs with unknown mechanisms of action. Future studies will be needed to further identify the transcriptomic and electrophysiological effects of both drugs in ET, possibly using more representative neuronal models such as iPSC-derived Purkinje cells, non-neuronal cell types as well as motor neurons.

## Methods

### Cell culture and drug treatment

The NPC line is from a healthy individual who is a Caucasian male of 62 years old and provided written informed consent. iPSCs were derived from fibroblasts and all the quality control criteria for validation of the iPSC line reprogramming and integrity have been performed. iPSC colonies were then cultured on Matrigel-coated dishes (BD Biosciences) using mTeSR1 medium (StemCell Technologies). Embryoid bodies were formed by mechanical dissociation of iPSC colonies to induce neural induction, using collagenase, and plating onto low-adherence dishes in STEMdiff™ Neural Induction Medium + SMADi (StemCell Technologies). Embryoid bodies were growing and maintained for 20 days in STEMdiff™ Neural Induction Medium + SMADi (StemCell Technologies). To obtain NPC, embryoid bodies were plated onto polyornithine/laminin (Sigma)-coated dishes in DMEM/F12 plus N2 and B27. Rosettes were manually collected and dissociated with accutase (Chemicon) after 1 week and plated onto Polyornithine/laminin-coated dishes in NPC media (DMEM/F12, 1× N2, 1× B27 (Invitrogen), 1 μg ml^−1^ laminin and 20 ng ml^−1^ FGF2 (Invitrogen)). The NPCs were then stained for the neural precursor marker (Nestin) and the pluripotency marker (Sox2) to confirm the NPC state. DAOYs (ATCC) cells were cultured as previously described^9^ in Eagle’s Minimum Essential Media (ATCC) supplemented with 10% FBS and 5% penicillin/streptomycin (Gibco). DAOYs and NPCs were treated for 5 days with 20 ng/ml of propranolol or 5 μg/ml of primidone (*n* = 3 per treatment/cell line). H2O- or DMSO (0.023%)-treated cells were used as controls for propranolol and primidone, respectively. Drug concentrations were chosen based on previous studies that tested efficient tremor-reducing serum levels of propranolol and primidone in ET patients^8,36^. A kill curve was used to determine lethal drug concentrations for DAOY cells and NPCs in culture (Supplementary Tables 10–12 and Supplementary Figs. 1 and 2).

### RNA-sequencing and differential expression analysis

RNA was extracted with the RNeasy Mini Kit (Qiagen). cDNA library preparation was done using NEBNext rRNA Depletion Kit with random hexamer cDNA generation (New England Biolabs). Samples were sequenced on the Illumina NovaSeq6000 platform (150 bp paired-end reads, 150 M reads). FASTQ files were pseudo-aligned to the Ensembl v102 annotation of the human genome using Salmon v1.4.0^37^. Gene-level differential expression analysis was done using the R package Sleuth^12^. Only genes with a minimum of 10 scaled reads per base in 90% of samples were kept to filter out low-count genes. Cell types and treatments were analyzed separately using the WT. The full model for the WT was:

1 $${\rm{Differentially}}\,{\rm{expressed}}\,{\rm{genes}}\,({\rm{DEG}})\sim {\rm{plate}} + {\rm{buffer}} + {\rm{treatment}}$$

MA plots and *p* value histograms displayed expected distributions (Supplementary Figs. 3 and 4). Meta-analysis of gene *Z*-scores was performed to analyze convergent DEG across cell types and treatments. Briefly, *Z*-scores for each gene were calculated and then summed across different combinations of cell types and treatments using Stouffer’s *Z* method^38^. Multiple analyses were performed notably propranolol specific effect across cell types (labeled “prop”; Supplementary Table 4), primidone effect across cell types (“prim”; Supplementary Table 5), convergent propranolol and primidone effect in each cell type (“daoy” and “npc”; Supplementary Tables 8 and 9 respectively) and convergent primidone and propranolol effects across both cell types (“all”; Supplementary Table 4). False discovery rate was controlled for using the Benjamini-Hochberg procedure (*q* value threshold <0.05). *TRAPPC11* Log2FC was validated in both propranolol-treated DAOYs and NPCs (Supplementary Table 13). At least three DEGs with highest Log2FC per condition were validated using TaqMan qPCR probes as well as 4 top convergent DEGs with 3 out of 4 Log2FCs in the same direction (*ROBO1, FAT4, ZMIZ1* and *MAP1B*) (Supplementary Table 13).

### WGCNA, co-expression and pathway enrichment

WGCNA was done using the R package^39^. DAOY and NPC sequencing results were analyzed separately, merging both primidone and propranolol treatments in the analysis. Normalized TPM values obtained from Sleuth (“sleuth_to_matrix”) were used for the analysis. To filter out noisy low-count genes, only genes with a minimum of 10 TPM in 47% of samples were kept, for a final list of 8549 genes in DAOYs and 9260 genes for NPC. Two outlier samples (“DAOY_PRIM_03” and “NPC_PRIM_02”) were removed from the analysis based on sample clustering dendrogram for a final 22 samples in our WGCNA analysis. Fisher’s exact test was used to calculate gene-module *p* values. Co-expression analysis was performed using GeneNetwork2.0^18^. Pathway enrichment analysis was done using the gprofileR R package^40^. Briefly, gene-lists were made from convergent DEGs across multiple conditions (both drugs in DAOYs or NPCs, propranolol or primidone in both cells, both drugs in both cells). Custom background used in gprofiler comprised genes expressed in either DAOYs, NPCs or both when pertinent. The g:SCS algorithm was used for multiple testing correction (*q* value threshold <0.1).

### Correlation with ET TWAS summary statistics

ET transcriptome wide-association studies (TWAS) summary statistics were obtained from Liao et al.^21^. A linear model was used to measure the strength of association between gene-level drug *Z*-scores and TWAS *Z*-scores, controlling for gene length and gene GC content (“lm” function in R). Weighted *Z*-scores were also used to account for significance of effect. The formula used were:

2 $${\rm{TWAS}}.Z = {\rm{Drug}}.Z + {\rm{Gene}}\,{\rm{length}} + {\rm{GC}}\,{\rm{content}}$$

And for the weighted *Z*-score analysis, given by:

3 $${\rm{TWAS}}.Z^2 = {\rm{Drug}}.Z^2 + {\rm{Gene}}\,{\rm{length}} + {\rm{GC}}\,{\rm{content}}$$

Both an unweighted and weighted *Z*-score test was used to assess the correlation with TWAS summary statistics. The weighted test allows to weigh *Z*-scores by their significance, akin to subsetting for only those with FDR < 0.05 but retains direction and size effect information (upregulated (positive *Z*-scores) or downregulated (negative *Z*-scores) gene expression). For example, a small non-significant *Z*-score (*Z* = 1), will result in a small *Z*-score after weighing (e.g., *Z* * (absolute *Z*) = 1*1 = 1). A large *Z*-score (*Z* = 4) will result in a larger significant *Z*-score after weighing (e.g., *Z* * (absolute Z) = 4 *4 = 16). This permits a more holistic approach to testing for correlations between DEGs across TWAS and the drug screen as opposed to subsetting DEGs based on direction and significance. Association *p* values were corrected for multiple testing using Benjamini-Hochberg (*q* value threshold <0.05).

### Single-cell enrichment analysis

A one sample *Z*-test was used to test enrichment of drug-targeted genes as described previously^22^. An ET gene-set was curated from genes associated with ET from linkage, whole-exome, GWAS and transcriptomic studies^2,9^. Drug gene-sets were made from convergent DEGs (FDR < 0.05) across different conditions (DAOY, NPC, propranolol, primidone, all conditions). Adult cerebellum single-nucleus RNA-sequencing data was obtained from Lake et al. (GEO accession: GSE97930)^23^. Average cell counts per cell type were obtained using Seurat v4.0.1^41^. Trimmed means per cell type from adult cortex single-cell RNA-sequencing were obtained from the Allen Brain Atlas Smart-seq multiple cortical regions dataset^42^. To account for drop-out rates and reduce zero-inflation of the single-cell count matrices, low average count genes were filtered out in both cerebellum (<0.5 counts in 7/10 cell types) and cortex (<1 count in 85/121 cell types). Single sample *Z*-tests were used to obtain cell type specific enrichment *Z*-scores:

4 $$Z-{\rm{score}} = \frac{{{\rm{Mean}}\,{\rm{geneset}}\,{\rm{counts}} - {\rm{Mean}}\,{\rm{cell}}\,{\rm{type}}\,{\rm{expression}}\,{\rm{counts}}}}{{{\rm{Geneset}} = {\rm{standard}}\,{\rm{deviation}}\, \times\, \sqrt {{\rm{Number}}\,{\rm{of}}\,{\rm{genes}}\,{\rm{in}}\,{\rm{geneset}}} }}$$

### Loss-of-function analysis

The distribution of mutational constraint scores for drug DEGs was assessed using pLoF o/e ratio scores obtained from gnomAD^30^. pLof scores for convergent genes across all conditions with *q* val <0.05 were compared all protein coding genes passing QC from the Sleuth differential expression analysis. To account for coding sequence length and gene GC percentage, propensity score matching with replacement was used (matchIT package in R^43^) to measure pLoF score distribution differences between DE drug genes and all protein coding genes included in the meta-analysis. Nearest neighbor matching with the maximum number of matches (ratio = 1:43) between non-DEGs and DEGs was used. A Wilcoxon unpaired test was done on the matched data. The same methods were used to assess pLoF score differences of upregulated (match ratio = 1:57) and downregulated (match ratio = 1:178) DEGs with all protein coding genes.

### Statistical tests

No statistical methods were used to determine sample sizes prior to experiments. A randomized layout was used when treating both cell types with propranolol and primidone as well as during RNA-sequencing. Statistical tests were performed in R version 4.0.2 and Rstudio version 1.4.11061. Differential expression analysis was done using the WT in Sleuth. Fisher’s exact test was used for WGCNA as well as pathway enrichment analysis using g:profileR. A Wilcoxon unpaired test was used to compare pLoF scores of DEGs. A one sample *z*-score was used to assess enrichment of DEGs in single-cell and single-nucleus data.

### Reporting summary

Further information on research design is available in the Nature Research Reporting Summary linked to this article.

## Supplementary information

Supplementary Dataset

Supplementary Figures

Reporting Summary

## Data Availability

Raw RNA-seq data are available on SRA (PRJNA857077) and upon request. Single-cell datasets are available through the Allen Brain Atlas (Smart-seq multiple cortical areas dataset) and GEO (GSE97930).

## Code availability

Code is available upon reasonable request. Packages and software used are described in the “Methods” section.
