# Supplementary material for: Transcriptomic effects of propranolol and primidone converge on molecular pathways relevant to essential tremor
Source: NPJ Genom Med. 2022 Aug 4;7:46. doi: 10.1038/s41525-022-00318-9 (PMC9352876; doi:10.1038/s41525-022-00318-9)
Supplement: Supplementary file 2 — Supplementary Figures [file 41525_2022_318_MOESM2_ESM.docx]

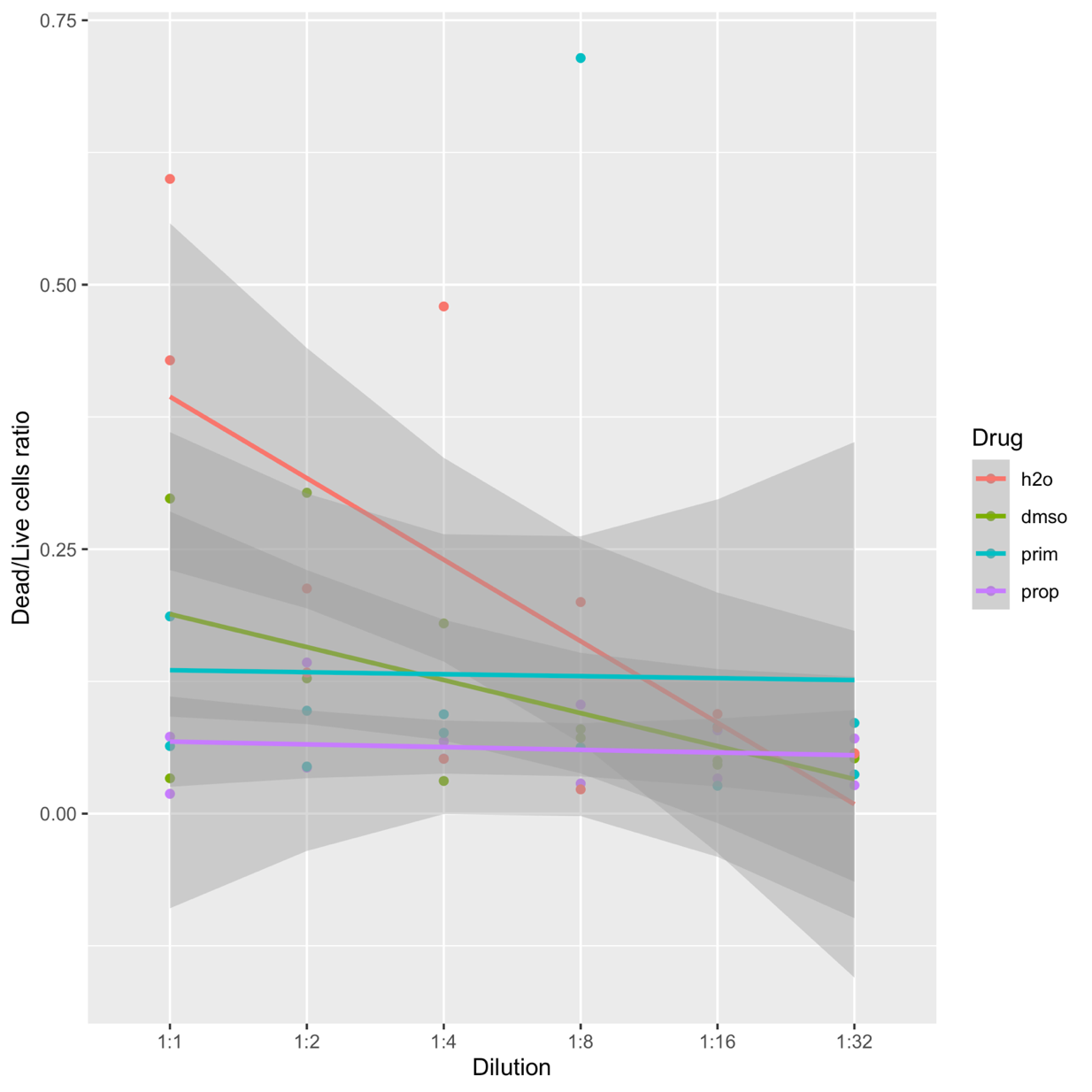


**Supplementary Figure 1. DAOY kill curve.** Dead over live cell ratios were calculated based on NucGreen and NucBlue (DAPI) staining after 5 days of treatment. Dilutions are calculated from initial concentrations of drugs or DMSO (%; corresponding to the percentage of DMSO that primidone was diluted in). 1:1 dilution; Propranolol = 0.0156 μg/mL, Primidone = 25 μg/mL; DMSO = 0.235%.

**
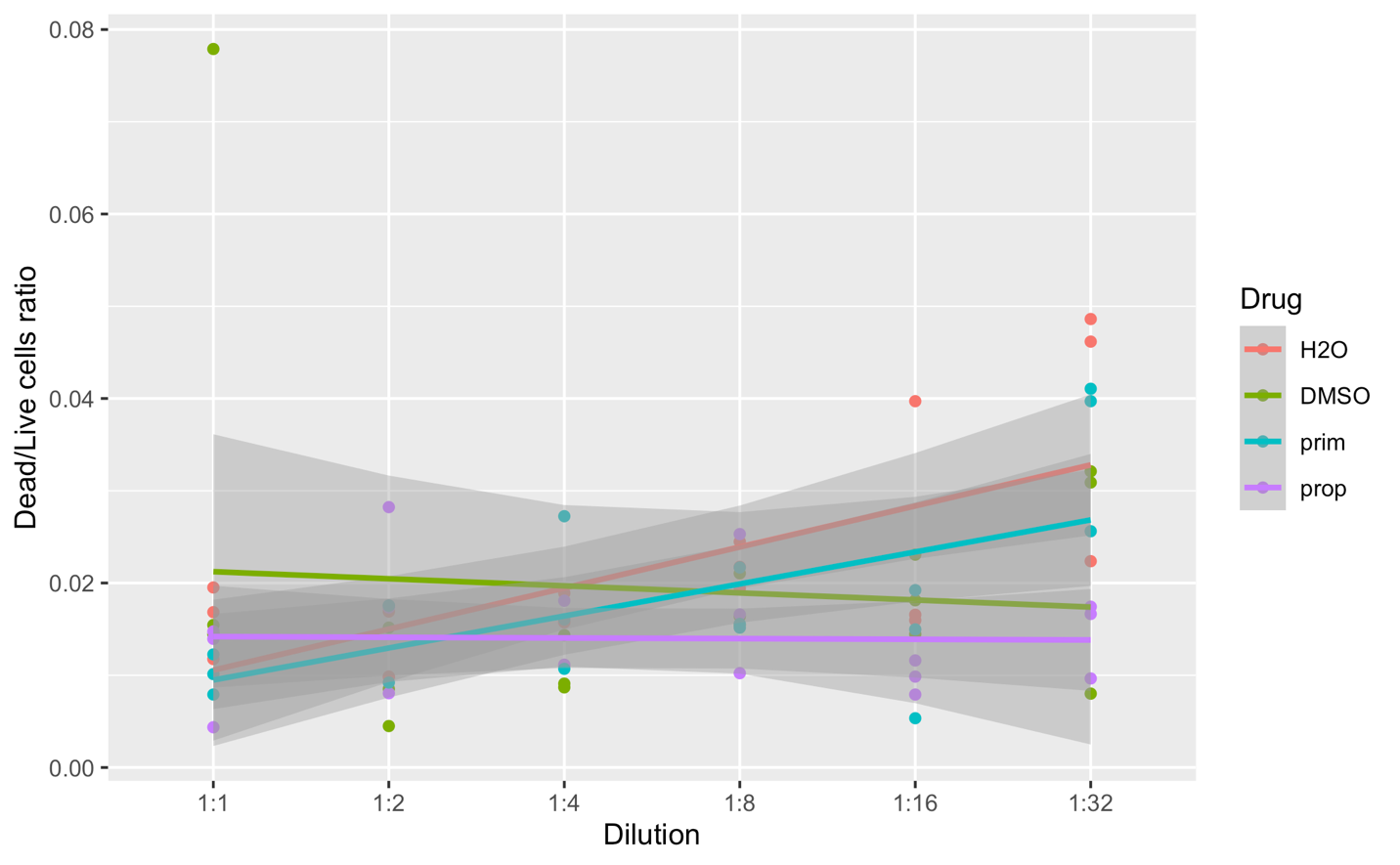
**

**Supplementary Figure 2. NPC kill curve.** Dead over live cell ratios were calculated based on NucGreen and NucBlue (DAPI) staining after 5 days of treatment. Dilutions are calculated from initial concentrations of drugs or DMSO (%; corresponding to the percentage of DMSO that primidone was diluted in). 1:1 dilution; Propranolol = 0.0156 μg/mL, Primidone = 25 μg/mL; DMSO = 0.235%.

**
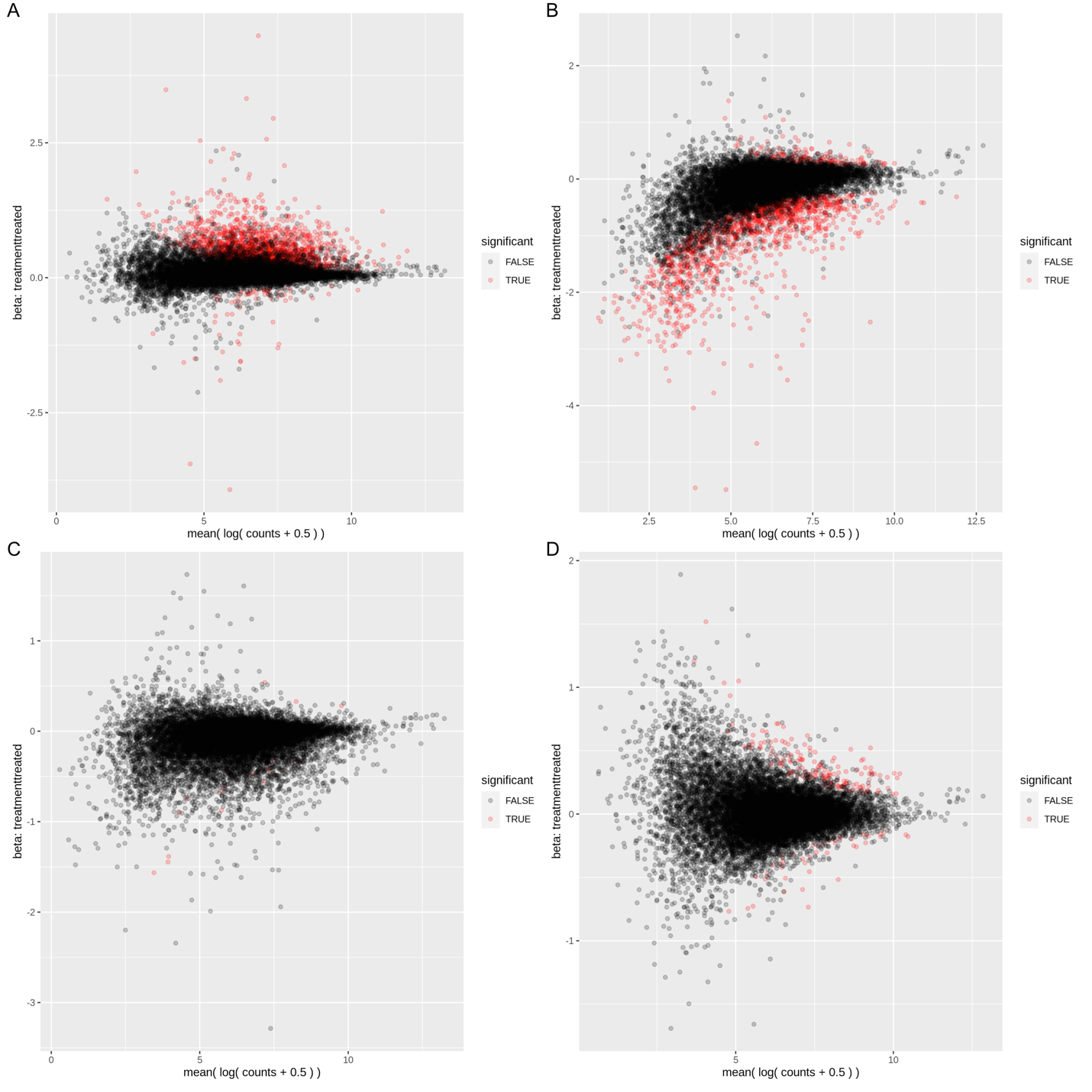
**

**Supplementary Figure 3. Mean A plots.** A. DAOYs treated with propranolol. B. DAOYs treated with primidone. C. NPCs treated with propranolol. D. NPCs treated with primidone


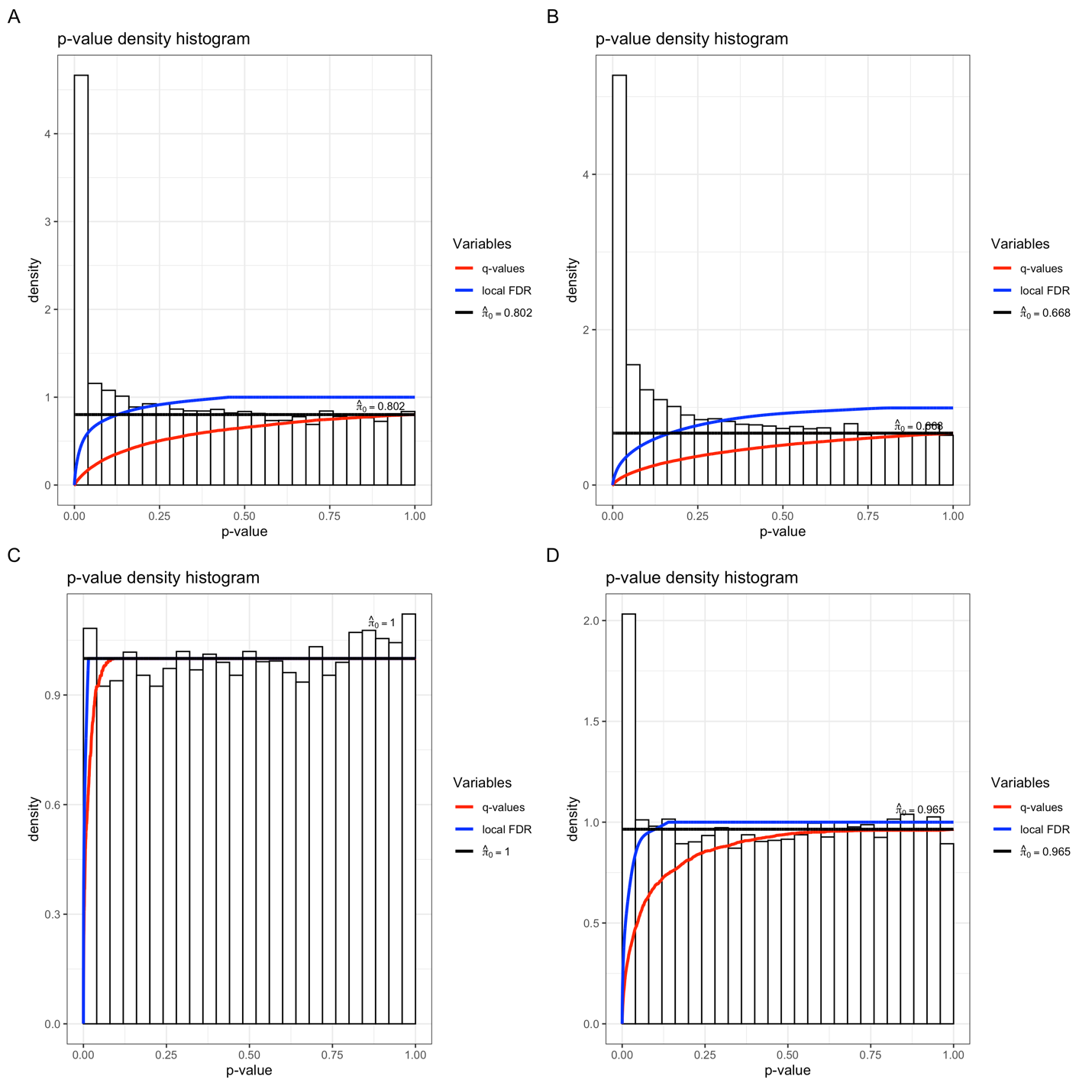


**Supplementary Figure 4. P-value histograms.** A. DAOYs treated with propranolol. B. DAOYs treated with primidone. C. NPCs treated with propranolol. D. NPCs treated with primidone
